# Supplementary figures and images for: BRD2 regulation of sigma-2 receptor upon cholesterol deprivation
Source: Life Sci Alliance. 2020 Nov 24;4(1):e201900540. doi: 10.26508/lsa.201900540 (PMC7723276; doi:10.26508/lsa.201900540)

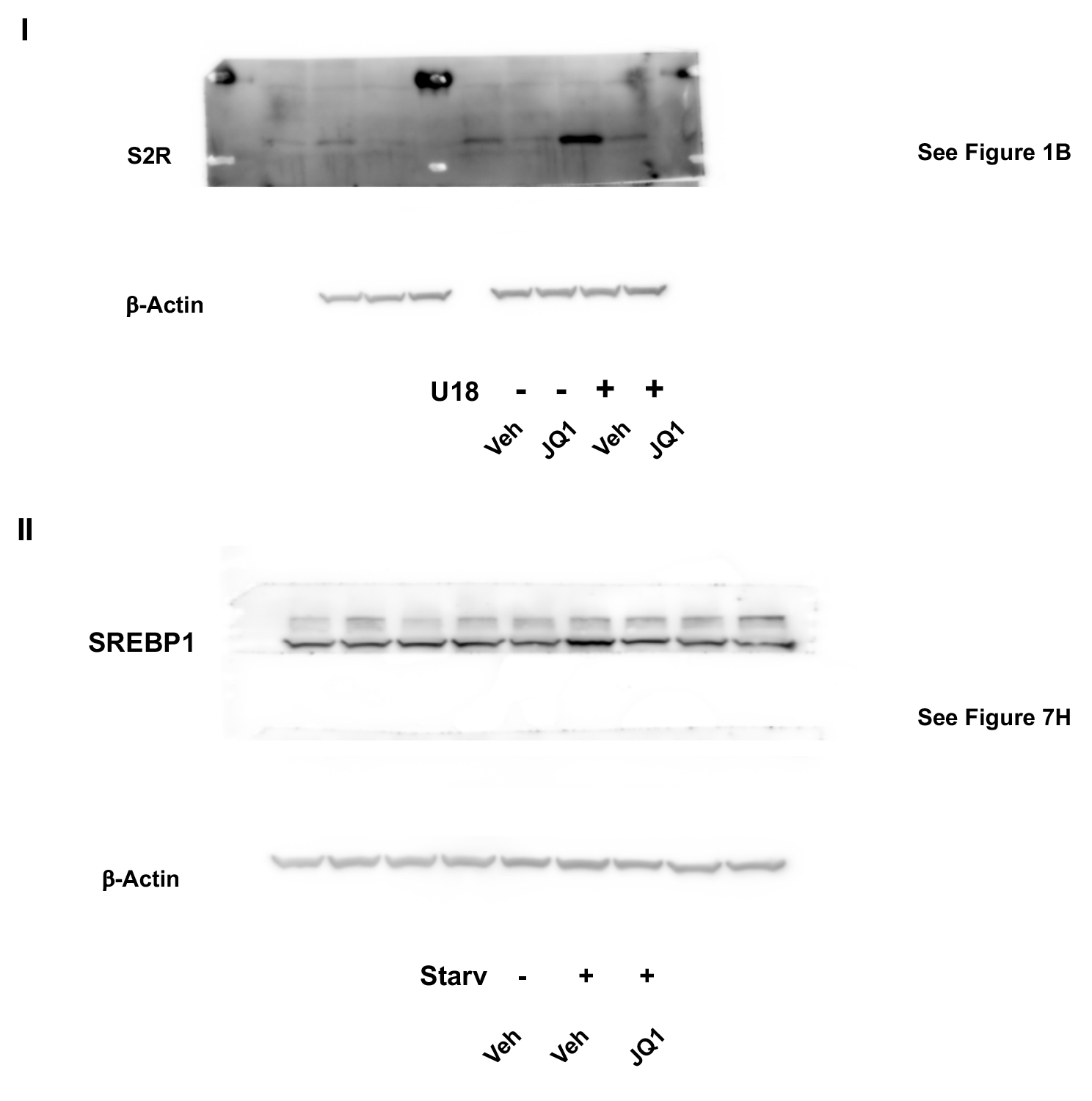

Supplement: Supplementary file 1 [file LSA-2019-00540_SdataF1_F7.tif]

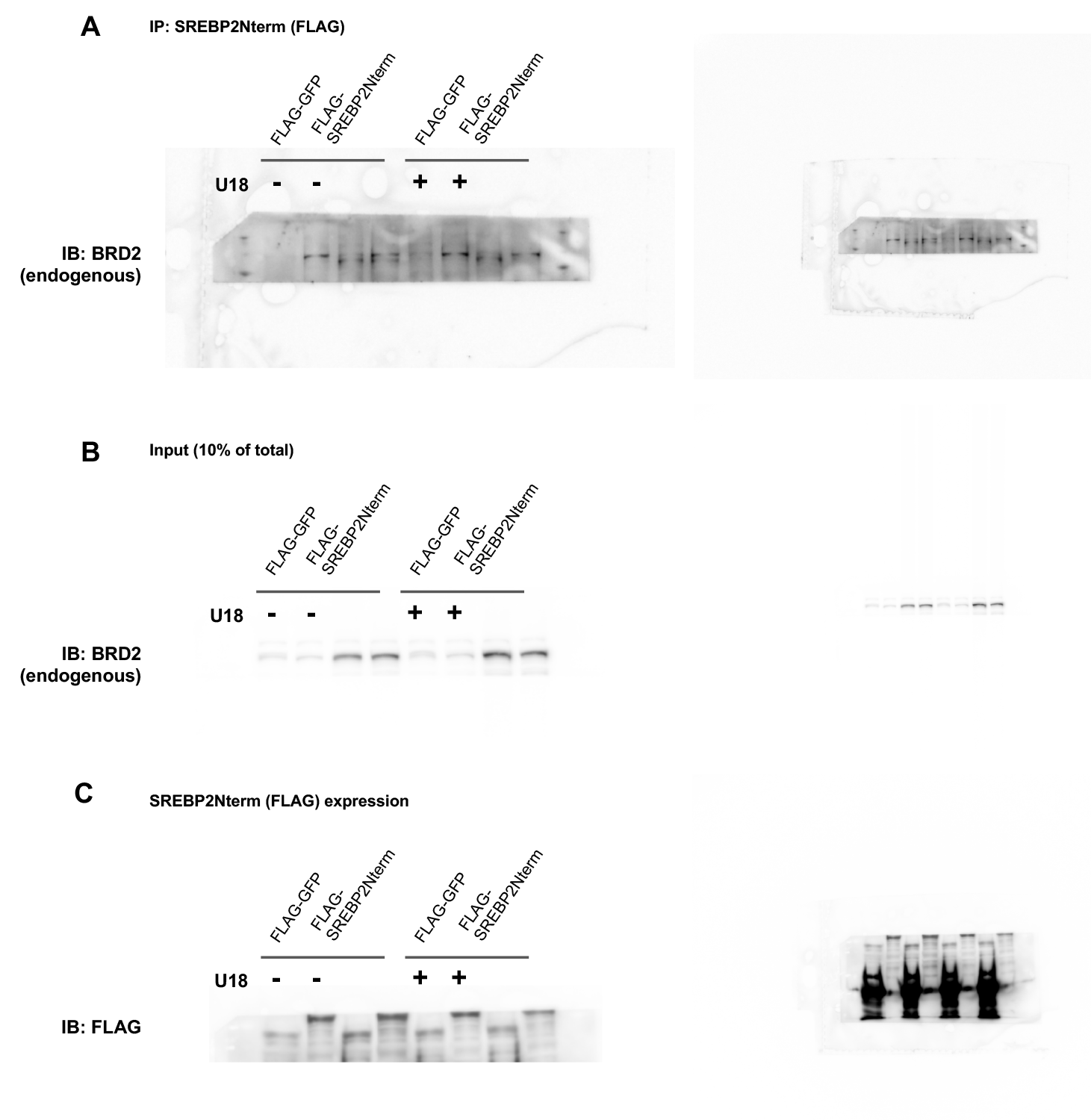

Supplement: Supplementary file 2 [file LSA-2019-00540_SdataF9.tif]
