# Supplementary material for: BRD2 regulation of sigma-2 receptor upon cholesterol deprivation
Source: Life Sci Alliance. 2020 Nov 24;4(1):e201900540. doi: 10.26508/lsa.201900540 (PMC7723276; doi:10.26508/lsa.201900540)
Supplement: Supplementary file 3 [file LSA-2019-00540_TableS1.docx]

Supplemental Tables

Table S1. Primers used for qRT-PCR

|  | Forward | Reverse |
| --- | --- | --- |
| BRD2 | GAGCCTGGACATCAACAAAT | GCAGGAAAGGACATAGCG |
| BRD3 | ACGACATCATCAAGCACCCG | TTGGCAAACCTCATCTCAAACACG |
| BRD4 | GTGGGAGGAAAGAAACAGGGAC | AGGAGGATTCGGCTGAGGG |
| LXRα | GAGGTACAACCCTGGGAGTGAG | ATAGCAATGAGCAAGGCAAACT |
| LXRβ | GCTAACAGCGGCTCAAGAACTAAT | GCTCCGTGAAGTGGGCAAAG |
| NPC1 | CCTTGTCTGGGTACGTTTG | AGGGCAGTGGCGTTATT |
| NPC2 | GCGTCCCAGTTCCCTTTC | GTTGCCACTCCACCACC |
| S2R | CAGTGGTTTCAAGGGACAAAG | GAATGGGATGAGTAAGTAGGG |
| SCAP | GACTCTGACCGCAAACAAGG | GGGACAAAGGTGAACGAAATAC |
| SREBP1 | CTTTGCCGACCCTGGTGAGT | ATGGCGTTGTGGGCTGTGC |
| SREBP2 | ATTGTCCTGAGCGTCTTTGTG | CAGGCAGGTTTGTAGGTTGC |
| GAPDH | TGCACCACCAACTGCTTAGC | GGCATGGACTGTGGTCATGAG |
