# Supplementary material for: BRD2 regulation of sigma-2 receptor upon cholesterol deprivation
Source: Life Sci Alliance. 2020 Nov 24;4(1):e201900540. doi: 10.26508/lsa.201900540 (PMC7723276; doi:10.26508/lsa.201900540)
Supplement: Supplementary file 4 [file LSA-2019-00540_TableS2.docx]

Table S2. Oligonucleotide sequences for shRNAs

|  | Sense | Anti-sense |
| --- | --- | --- |
| BRD3-1 | CCGGGCTGATGTTCTCGAATTGCTACTCGAGTAGCAATTCGAGAACATCAGCTTTTTG | AATTCAAAAAGCTGATGTTCTCGAATTGCTACTCGAGTAGCAATTCGAGAACATCAGC |
| BRD3-2 | CCGGGAGATATGTCAAGTCTTGTTTCTCGAGAAACAAGACTTGACATATCTCTTTTTG | AATTCAAAAAGAGATATGTCAAGTCTTGTTTCTCGAGAAACAAGACTTGACATATCTC |
| BRD4-1 | CCGGCAGTGACAGTTCGACTGATGACTCGAGTCATCAGTCGAACTGTCACTGTTTTTTG | AATTCAAAAAACAGTGACAGTTCGACTGATGACTCGAGTCATCAGTCGAACTGTCACTG |
| BRD4-2 | CCGGCCTGGAGATGACATAGTCTTACTCGAGTAAGACTATGTCATCTCCAGGTTTTTG | AATTCAAAAACCTGGAGATGACATAGTCTTACTCGAGTAAGACTATGTCATCTCCAGG |
| SREBP2 | CCGGCCTCAGATCATCAAGACAGATCTCGAGATCTGTCTTGATGATCTGAGGTTTTTG | AATTCAAAAACCTCAGATCATCAAGACAGATCTCGAGATCTGTCTTGATGATCTGAGG |
| Scram-bled | CCGGCCTAAGGTTAAGTCGCCCTCGCTCGAGCGAGGGCGACTTAACCTTAGGTTTTTG | AATTCAAAAACCTAAGGTTAAGTCGCCCTCGCTCGAGCGAGGGCGACTTAACCTTAGG |
