# Supplementary material for: BRD2 regulation of sigma-2 receptor upon cholesterol deprivation
Source: Life Sci Alliance. 2020 Nov 24;4(1):e201900540. doi: 10.26508/lsa.201900540 (PMC7723276; doi:10.26508/lsa.201900540)
Supplement: Supplementary file 5 [file LSA-2019-00540_TableS3.docx]

Table S3. Antibodies for Western blotting

| Antibody | Company | Catalog number | Dilution |
| --- | --- | --- | --- |
| BRD2 | Proteintech | 22236-1-AP | 1:1000 |
| BRD3 | Proteintech | 11859-1-AP | 1:1000 |
| BRD4 | Abcam | Ab75898 | 1:1000 |
| SREBP1 | Santa cruz | Sc-13551 | 1:1000 |
| SREBP2 | BD Biosciences | 557037 | 1:200 |
| S2R | Novus | NBP1-30436 | 1:200 |
| FLAG | Sigma-aldrich | F3165 | 1:2000 |
| GAPDH | Cell Signaling Technologies | 2118S | 1:3000 |
| β -Actin | Abcam | ab6276 | 1:3000 |
