# Supplementary material for: BRD2 regulation of sigma-2 receptor upon cholesterol deprivation
Source: Life Sci Alliance. 2020 Nov 24;4(1):e201900540. doi: 10.26508/lsa.201900540 (PMC7723276; doi:10.26508/lsa.201900540)
Supplement: Supplementary file 6 [file LSA-2019-00540_TableS4.docx]

Table S4. Primers for ChIP-qPCR

|  |  | Forward primer | Reverse primer |
| --- | --- | --- | --- |
| S2R gene promoter | Region-1 (-5K bp from TSS) | ATAGAGGCAATTGGGGGCG | AATGCACCCACCCTTCT |
|  | Region-2 (-5K) | GGGGTAGGGTGGCCTTCAGCC | AATGCACCCACCCTTCT |
|  | Region-3 (-1K) | GCCCTCTGCTCGCCATCAAAG | GGAAGGAATGCAGCTTGGACT |
|  |  |  |  |
| SREBP2  gene  promoter | A region at -1K bp from TSS | GGGGAGGGACCTCACTATGC | AGCCAATGGGCGAGCGAAGC |
